# Impact of Environmental Factors and Biological Soil Crust Types on Soil Respiration in a Desert Ecosystem

**DOI:** 10.1371/journal.pone.0102954

**Published:** 2014-07-22

**Authors:** Wei Feng, Yuqing Zhang, Xin Jia, Bin Wu, Tianshan Zha, Shugao Qin, Ben Wang, Chenxi Shao, Jiabin Liu, Keyu Fa

**Affiliations:** Yanchi Research Station, College of Soil and Water Conservation, Beijing Forestry University, Beijing, China; University of Maryland, United States of America

## Abstract

The responses of soil respiration to environmental conditions have been studied extensively in various ecosystems. However, little is known about the impacts of temperature and moisture on soils respiration under biological soil crusts. In this study, CO_2_ efflux from biologically-crusted soils was measured continuously with an automated chamber system in Ningxia, northwest China, from June to October 2012. The highest soil respiration was observed in lichen-crusted soil (0.93±0.43 µmol m^−2^ s^−1^) and the lowest values in algae-crusted soil (0.73±0.31 µmol m^−2^ s^−1^). Over the diurnal scale, soil respiration was highest in the morning whereas soil temperature was highest in the midday, which resulted in diurnal hysteresis between the two variables. In addition, the lag time between soil respiration and soil temperature was negatively correlated with the soil volumetric water content and was reduced as soil water content increased. Over the seasonal scale, daily mean nighttime soil respiration was positively correlated with soil temperature when moisture exceeded 0.075 and 0.085 m^3^ m^−3^ in lichen- and moss-crusted soil, respectively. However, moisture did not affect on soil respiration in algae-crusted soil during the study period. Daily mean nighttime soil respiration normalized by soil temperature increased with water content in lichen- and moss-crusted soil. Our results indicated that different types of biological soil crusts could affect response of soil respiration to environmental factors. There is a need to consider the spatial distribution of different types of biological soil crusts and their relative contributions to the total C budgets at the ecosystem or landscape level.

## Introduction

Soil respiration (*R_s_*) accounts for the second largest carbon flux between terrestrial ecosystems and atmosphere, after gross primary productivity. Physical (e.g., soil temperature, moisture) and biological factors (e.g., microbial community) affecting *Rs* should be taken into consideration in order to accurately estimate global carbon balance [1]. However, we have limited knowledge on the biophysical controls of *R_s_* in dryland ecosystems. Drylands cover 41–47% of the terrestrial surface [2]. Biological soil crusts (BSCs) as a biological factor commonly cover 70% of the inter-canopy earth in dryland and are found in all ecosystems around the world [3]. BSCs consist of algae, lichen, moss, fungi, cyanobacteria, and bacteria and cover the top few millimeters of the soil surface [3], [4]. However, knowledge about the role of BSCs as a modulator of *R_s_* is still lacking [5]–[7]. It is important to study the effects of environmental factors, such as temperature and moisture, on *R_s_* under BSCs. This knowledge can reduce bias in ecosystem-level estimation of *R_s_* and can help us predict how climate changes will affect CO_2_ flux in desert ecosystems.

BSCs are an integral part of the soil system in arid regions worldwide [4]. *R_s_* studies in relation to BSCs have drawn much attention in the past decade [4]. In the Gurbantunggute desert, the mean *R_s_* of cyanobacteria/lichen-crusted soil is significantly higher than that of bare land after 15 mm rainfall [8]. In Kalahari sand, the CO_2_ flux of cyanobacteria-crusted soil is lower than that of disturbed crusted soil [6]. In the Iberian Peninsula, lichen-crusted soils are the main contributor to *R_s_* [9]. In the Mu Us desert, *R_s_* does not differ between BSC-dominated areas and bare land [10]. However, the limited knowledge about the role of BSCs as a modulator of *R_s_* on C cycle merely focused on particular species or communities. Although those have provided valuable insights on the effects of BSCs on C fluxes, in-situ data remain rare and we have incomplete understanding of the impact of different types of BSCs on *R_s_*.

Soil temperature (*T_s_*) and soil water content (*VWC*) are the key environmental factors responsible for variation in *R_s_* [11]. *T_s_* is the major control of *R_s_* through its influence on the kinetics of microbial decomposition, root respiration, and the diffusion of enzymes and substrate [12]. *VWC* controls the decomposition of soil organic matter, root respiration, and microbial actively [3], . *T_s_* and *VWC* were been predicted to increase at global scales in the following decades [2]. In order to assess the impact of the changing climate on ecosystem C flux, quantification of the effects of *T_s_* and *VWC* on *R_s_* is needed. Recent studies have shown that diurnal variations in *R_s_* are usually highly correlated with temperature of the surface soil layers [14], [15]. However, a few studies have reported a hysteresis effect and a decoupling between *R_s_* and soil surface temperature during drought conditions in boreal forests [16], tropical forests [17], Mediterranean ecosystems [18], and desert ecosystems [19]. Low water content may increase the degree of hysteresis between *R_s_* and *T_s_* [17], [18], [19] or, in some cases, may reduce it [20]. At the seasonal scale, *R_s_* is also highly correlated with changes in *T_s_* when water content is not limited [19], [21], [22]. Strong inhibition of *R_s_* has often been observed when soil water content is low [23]. All those are mainly focused on shrub soils or bare-land soils. However, our ability to capture the effects of environmental factors on *R_s_* in biologically-crusted soil is still lacking.

Understanding of how biologically-crusted soil types and environmental factors influence *R_s_* in a desert ecosystem, we measured *R_s_* in algae-, lichen-, and moss-crusted soil in the Mu Us Desert, northwestern China. The specific objectives of this study were: (1) to examine and compare the temporal variability of *R_s_* in three crusted soils; (2) to determine seasonal and diurnal patterns of *R_s_*; and (3) to assess the contributions of the three crusted soils to the amount of C released by *R_s_* at the ecosystem level.

## Materials and Methods

### 2.1 Ethics Statement

The study site is owned by Beijing Forestry University. The field work did not involve any endangered or protected species, and did not involve destructive sampling. Specific permits were required for the described study.

### 2.2 Site description

The research was conducted at the Yanchi Research Station (37°04′ to 38°10′ N and 106°30′ to 107°41′ E, 1550 m a.s.l.), Ningxia, northwest China. The area is located in the mid-temperate zone and characterized by a semiarid continental monsoon climate. The mean annual temperature is 8.1°C, the mean annual rainfall is 292 mm, 62% of which falls between July and September. The mean annual potential evaporation is 2100–2500 mm. All meteorological data were provided by the meteorological station of Yanchi County and represent 51 year averages (1954–2004). The vegetation in the area is dominated by *Artemisia ordosica*. The soil surface of inter-canopy is commonly covered by algae, lichen, and moss crusts, which are mainly composed of *Microcoleus vaginatus*, *Oscillatoria chlorine*, *Collema tenax*, and *Byumargenteum*, respectively [10], [24]. The physical and chemical characteristics of the three crusted soils are shown in Table 1. The soil of the area is aripsamment with 1.61 g cm^−3^ in soil bulk density.

**Table 1:** Physical and chemical characteristics of BSC layer in the study sites [41], [42].

Soil type	SOC (%)	TNC (%)	SBD (g·cm^−3^)	pH	Particle content (<0.05 mm)(%)
Algae-crusted soil	0.34±0.13	0.02±0.01	1.69±0.10	8.81±1.40	6.16±1.14
Lichen-crusted soil	1.33±0.09	0.07±0.01	1.60±0.03	8.62±1.10	8.43±1.41
Moss-crusted soil	2.14±0.19	0.10±0.02	1.70±0.45	7.84±1.60	11.07±0.81

SOC: soil organic carbon; TNC: total nitrogen content; SBD: soil bulk density.

### 2.3 Soil respiration measurements

Continuous measurements of soil surface CO_2_ efflux (*R_s_*) were made in an open area at *Artemisia ordosica* shrub land with intact algae, lichen and moss crusts between June and October in 2012. An automated soil respiration system (Model LI 8100A fitted with a LI-8150 multiplexer, LI-COR, Nebraska, USA) was used to measure *R_s_*. Three permanent PVC collars (20.3 cm in diameter, 10 cm in height, inserted ∼7 cm) were separately installed in intact algae-, lichen- and moss-crusted soil in March 2012, three months before the start of measurements. A permanent opaque chamber (model LI-104, LI-COR, Nebraska, USA) was set on each collar. The measurement time for each chamber was 3 min and 15 s, including a 30 s pre-purge, a 45 s post-purge, and a 2 min observation period. Hourly *T*_s_ and *VWC* at 5-cm depth were measured near the chamber using an 8150-203 temperature sensor and an EC_H2O_ soil moisture sensor (Li-COR, Nebraska USA), respectively. During observation, any plants re-growing within collars were manually removed. Rainfall was measured near the chamber by a manual rain gauge and a tipping-bucket rain gauge (model TE525MM, Campbell Scientific, UT, USA). Half-hourly incident photosynthetically active radiation (PAR) was measured using a quantum sensor (PAR-LITE, Kipp & Zonen, The Netherlands) near the chambers.

### 2.4 Data treatment and analysis

**Figure 1:**
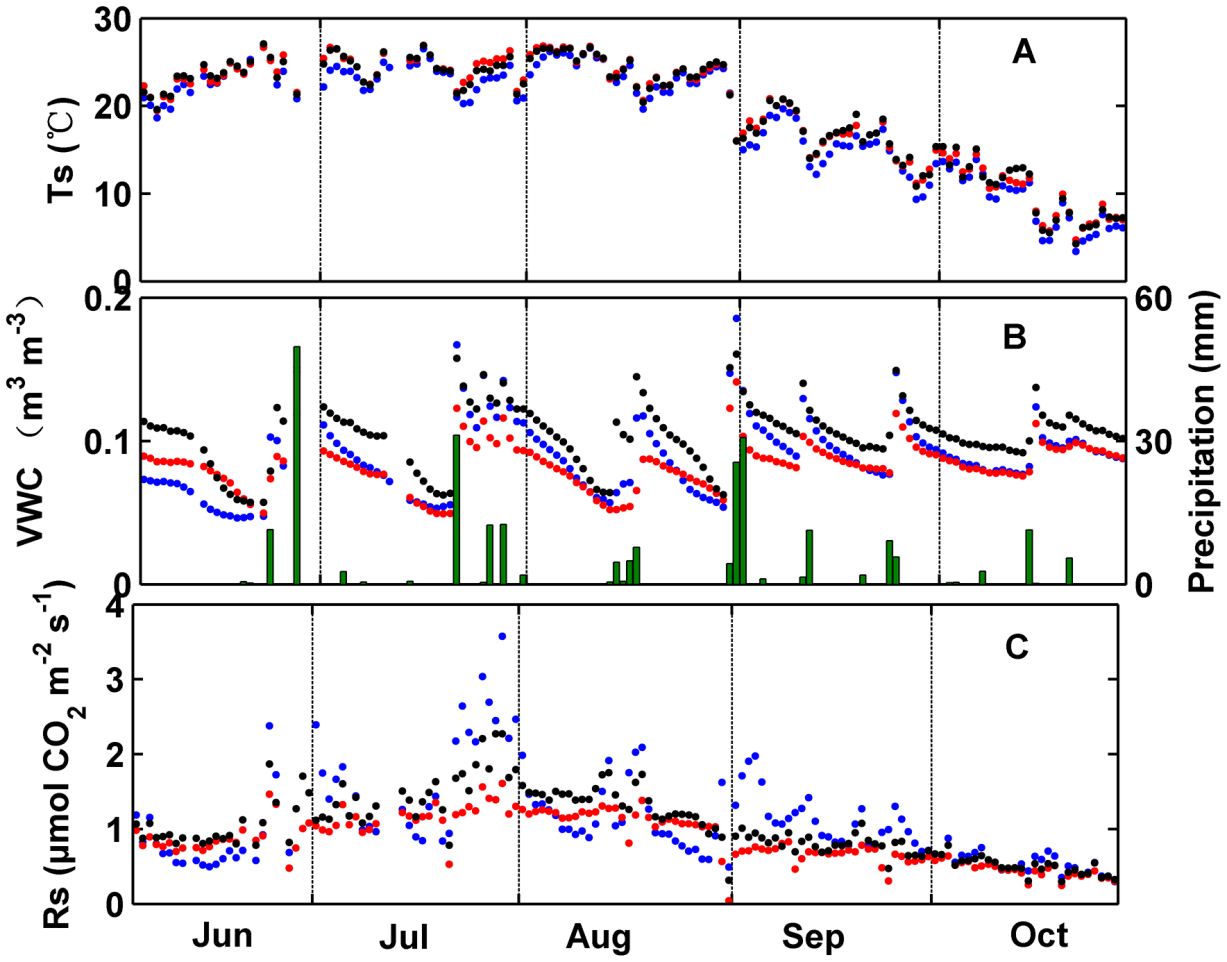
Daily mean of soil respiration (*R_s_*), soil temperature (*T_s_*), and soil volumetric water content (*VWC*) in soil crusted with algae (red), lichen (black), and moss (blue).

The CO_2_ efflux values greater than 15 µmol m^2^ s^−1^ or less than -1 µmol m^2^ s^−1^ were considered abnormal and removed from the dataset. Instrument failure, sensor calibration, and poor-quality measurements together resulted in the loss of 4% to 5.4% of the values for three chambers from June to October 2012 (Fig. 1).

To avoid including the impacts of photosynthesis and Birch effects on the seasonal responses of *R_s_* to *T_s_* and *VWC*, certain observations were removed from the dataset. (1) Daytime (photosynthetically active radiation, PAR >5 µmol m^−2^ s^−1^) CO_2_ efflux values were removed to ensure that no photosynthesis effects were included. (2) Measurements recorded immediately (within 30 min) after a rain event were excluded because they were potentially affected by the rewetting of the upper soil layers, which could stimulate respiration [25], [26]. The daily mean nighttime value (*R_s_*, *T_s_*, and *VWC*) was computed as the average of the hourly values when PAR was below 5 µmol m^−2^ s^−1^. Daily mean nighttime values were used to examine the seasonal responses of *R_s_* to *T_s_* and *VWC*. The seasonal relationships between *R_s_* and *T_s_* were estimated using four common models: Exponential (*Q*_10_), Arrhenius, Quadratic, and Logistic (see Table 2). The four models were fitted separately for each crusted soil. Root mean square error (RMSE) and the coefficient of determination (*R^2^*) were used to evaluate model performance. Temperature-normalized daily mean nighttime *R_s_* (*R*_sN_), calculated as the ratio of the observed nighttime *R_s_* to the value predicted by the *Q*_10_ model, was used to analyze the seasonal dependence of daily mean nighttime *R_s_* on *VWC*. Three bivariate models with *T_s_* and *VWC* as independent variables were developed to show the combined effect of both variables (Table 3).

**Table 2:** Parameters and statistics for the analysis of the dependence of daily mean nighttime *R*_s_ (µmol m^−2^ s^−1^) on daily mean nighttime *T_s_* (°C) at 5-cm depth when daily mean nighttime *VWC* (m^3^ m^−3^) was above and below 0.075 m^3^ m^−3^ in algae-and moss-crusted soil, and 0.085 m^3^ m^−3^ in lichen-crusted soil.

Soil Type	Model	*VWC* >0.075 m^3^ m^−3^					*VWC* <0.075 m^3^ m^−3^				
		*a*	*b*	*c*	*Adj.R^2^*	RMSE	*a*	*b*	*c*	*Adj.R^2^*	RMSE
Algae-crusted soil	*Q*_10_	0.38	2.01		**0.82**	0.1254	0.55	1.52		0.57	0.1482
	Quadratic	0.0014	−0.002	0.25	**0.82**	0.1262	−0.01	0.68	−7.67	0.52	0.1511
	Logistic	32.2	0.07	71.79	**0.82**	0.1262	1.10	0.51	19.28	0.57	0.1513
	Arrhenius	0.38	0.0005		**0.82**	0.1255	0.54	0.00031		0.57	0.1482
Moss-crusted soil	*Q*_10_	0.55	1.97		**0.53**	0.377	0.32	1.81		0.08	0.2446
	Quadratic	0.00053	0.06	−0.01	**0.53**	0.3708	0.01	−0.45	5.158	0.10	0.2419
	Logistic	1.52	0.19	13.58	**0.53**	0.3639	3.60	0.026	79.02	0.0006	0.2545
	Arrhenius	0.55	0.00047		**0.53**	0.3759	0.32	0.00043		0.076	0.244

Type	Model	*VWC*>0.085 m^3^ m^−3^					*VWC*<0.085 m^3^ m^−3^				
		*a*	*b*	*c*	*Adj.R^2^*	RMSE	*a*	*b*	*c*	*Adj.R^2^*	RMSE
Lichen-crusted soil	*Q*_10_	0.46	2.13		**0.74**	0.2196	0.43	2.00		0.062	0.2849
	Quadratic	0.002	0.007	0.22	**0.74**	0.2198	−0.07	3.30	−39.6	0.12	0.2759
	Logistic	3.31	0.10	27.95	**0.74**	0.2198	1.26	1.33	21.65	0.092	0.2803
	Arrhenius	0.46	0.00053		**0.74**	0.2191	0.41	0.00051		0.063	0.2848

*Q*_10_: *R_s_* = *ab* ^(*T*^ *_s_* ^-10)/10^; Arrhenius: *R_s_* = *a*exp(*b*/283.15 8.314)(1-283.15/*T_s_*); Quadratic: *R_s_* = *a*·*T_s_* ^2^+*b*·*T_s_*+c; Logistic: *R_s_* = *a*/(1+exp(*b*(*c*-*T_s_*); *Q*_10_: relative increase in *R_s_* for a 10°C increase in *T_s_*; *Adj.R* ^2^ is the adjusted coefficient of determination; RMSE is the root-mean-square error; *a*, *b*, and *c* are fitted parameters; values in bold indicate best fits according to *Adj*.*R^2^* and RMSE.

**Table 3:** Parameters, statistics, and predicted values from temperature-only and bivariate models of soil respiration on the basis of daily mean values.

Soil Type	Model	*a*	*b*	*c*	*d*	*Adj.R^2^*	RMSE	Predicted *R_s_* (g C m^−2^)
Algae-crusted soil	*Q*_10_	0.38	1.98			0.82	0.1293	123.22
	*Q*_10_-power	0.53	2.03	0.13		0.82	0.1268	127.46
	*Q*_10_-linear	1.76	1.99	0.13	0.21	0.82	0.1262	126.65
	*Q*_10_-hyperbolic	2.042	−0.13	3.83	0.015	**0.83**	0.1268	**126.21**
Lichen-crusted soil	*Q*_10_	0.48	1.98			0.68	0.2393	155.92
	*Q*_10_-power	1.34	2.20	0.53		0.74	0.2151	132.43
	*Q*_10_-linear	1.19	2.07	0.68	0.32	0.72	0.7129	158.84
	*Q*_10_-hyperbolic	2.167	−0.62	6.86	0.036	**0.76**	0.2066	**165.39**
Moss-crusted soil	*Q*_10_	0.56	1.54			0.22	0.4127	130.43
	*Q*_10_-power	12. 61	2.07	1.36		0.67	0.2699	146.92
	*Q*_10_-linear	1.43	1.82	1.72	0.19	0.43	0.3522	134.57
	*Q*_10_-hyperbolic	2.08	−0.51	9.31	0.013	**0.67**	0.2691	**147.08**

*Q*_10_-power: *R_s_* = *a*·*b* ^((*T*^ *_s_* ^-10)/10)^ *VWC^c^*. *Q*_10_-linear: *R_s_* =  *a*·*b* ^((*T*^ *_s_* ^-10)/10)^ (*c* *VWC*+*d*). *Q*_10_-hyperbolic: *R_s_* = *a* ^((*T*^ *_s_* ^-10)/10)^·(*b*+*c*·*VWC*+*d*/*VWC*); *Adj.R^2^* is the adjusted coefficient of determination; RMSE is the root-mean-square error; *a*, *b*, and *c* are fitted parameters; values in bold indicate best fits according to *Adj*.*R^2^* and RMSE.

To ensure that the measurements of diurnal responses of *R_s_* to *T_s_* and *VWC* were not affected by photosynthesis, CO_2_ flux measurements taken within two days after a significant rain event (>10 mm) were removed from the dataset. Field observation revealed that the water content of BSCs layers decreased to the water compensation point of photosynthesis within two days after the last significant rain event (>10 mm) in all three crusted soils [24], [27]. The mean diurnal courses of *R_s_*, *T_s_*, and *VWC* were computed for each month by averaging the hourly means for each time of day. Cross-correlation analysis was used to detect hysteresis between *R_s_* and *T_s_* at the diurnal scale. Correlation analysis was used to evaluate the relationship between *R_s_* and *T_s_* (Table 4). All analyses were processed in Matlab 7.11.1 (R2010b, the Mathworks Inc., Natick, MA, USA).

**Table 4:** Correlation and hysteresis analysis of monthly diurnal courses of soil respiration (*R_s_*) and soil temperature (*T_s_*) at 5-cm depth.

Soil Type		Jun		Jul		Aug		Sep		Oct	
					Non-Synchronized						
		*r*	*P*	*r*	*P*	*r*	*P*	*r*	*P*	*r*	*P*
*T_s_*-*R_s_*	Algae-crusted soil	0.231	0.279	0.521**	0.009	0.441*	0.031	0.571**	0.001	0.438*	0.032
	Lichen-crusted soil	0.435*	0.034	0.728**	0.001	0.591**	0.002	0.625**	0.01	0.405*	0.05
	Moss-crusted soil	−0.210	0.362	0.531**	0.008	0.208	0.331	0.668**	0.001	0.212	0.781
					Synchronized						
		*r*	*P*	*r*	*P*	*r*	*P*	*r*	*P*	*r*	*P*
*T_s_*-*R_s_*	Algae-crusted soil	0.844	0.001	0.943	0.001	0.914	0.001	0.962	0.001	0.971	0.001
	Lichen-crusted soil	0.701	0.001	0.875	0.001	0.658	0.001	0.952	0.001	0.917	0.001
	Moss-crusted soil	0.932	0.001	0.903	0.001	0.851	0.001	0.966	0.001	0.781	0.0001
Lag time	Algae-crusted soil	2		3		2		3		4	
	Lichen-crusted soil	3		2		1		3		3	
	Moss-crusted soil	5		3		3		3		4	

*r* is the Pearson correlation coefficient; *P* is the significance level.

To examine whether daily mean nighttime *R_s_*, *T_s_*, and *VWC* differed among biologically-crusted soils, we used a two-way (biologically-crusted soil types and time) ANOVA, with repeated measures of one of the factors (time). The environmental factors show relatively small variation within three days. Thus, we selected consecutive three-day periods as the three replication for statistical requirements. When significant biologically-crusted soils effects were found (*P*<0.05), the Tukey HSD post hoc test was employed to evaluate differences between biologically-crusted soil types. Prior to these analyses, data were tested for assumptions of normality and homogeneity of variances and were log-transformed when necessary. All the ANOVA analyses were performed using the SPSS 15.0 statistical software (SPSS Inc., Chicago, Illinois, USA).

## Results

### 3.1. Hysteresis between R_s_ and T_s_

**Figure 2:**
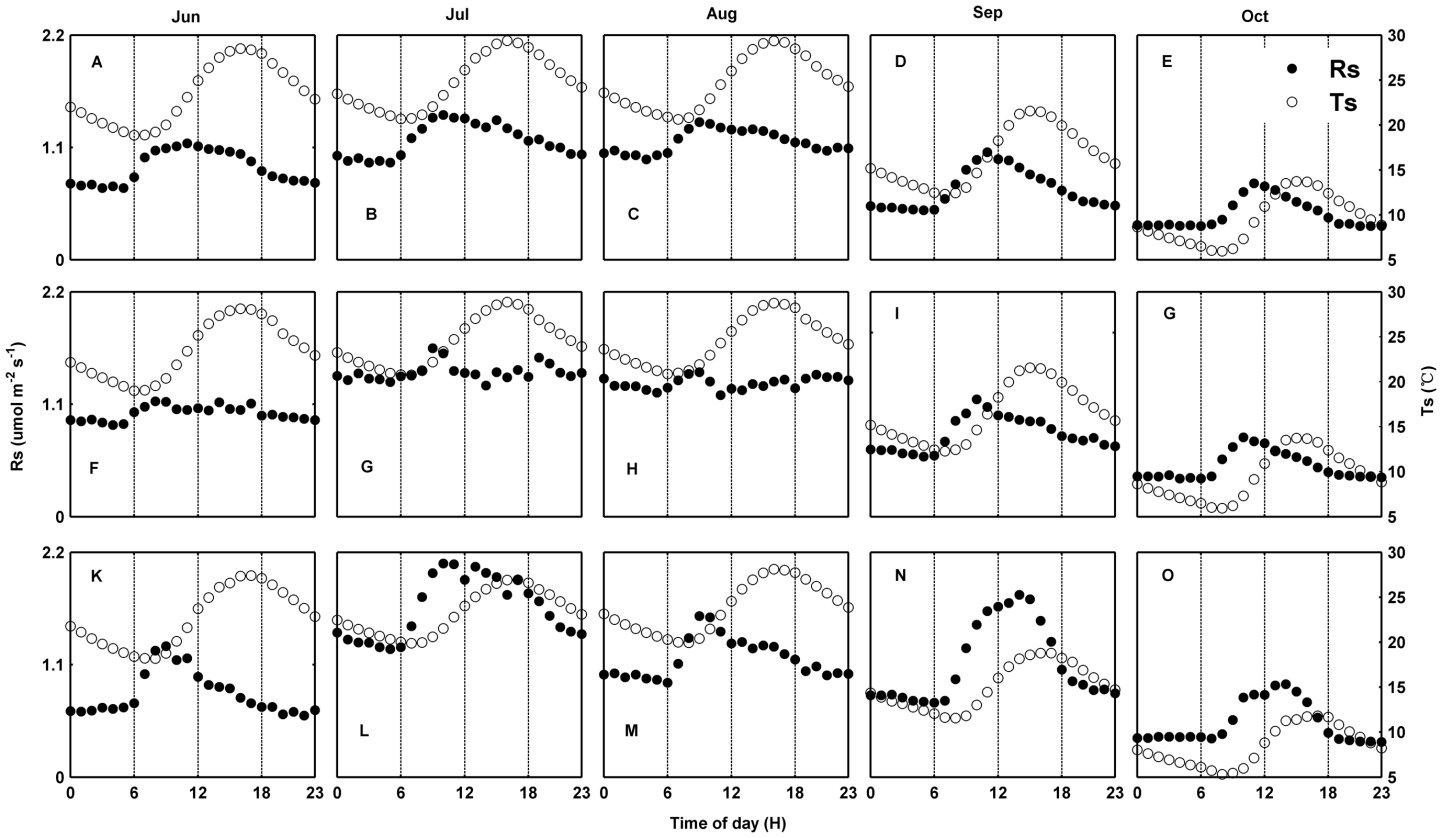
Monthly diurnal courses of soil respiration (*R_s_*) and soil temperature (*T_s_*) in soil crusted with algae (A-E), lichen (F-J), and moss (K-O). Each point is the monthly mean for a particular time of day.

**Figure 3:**
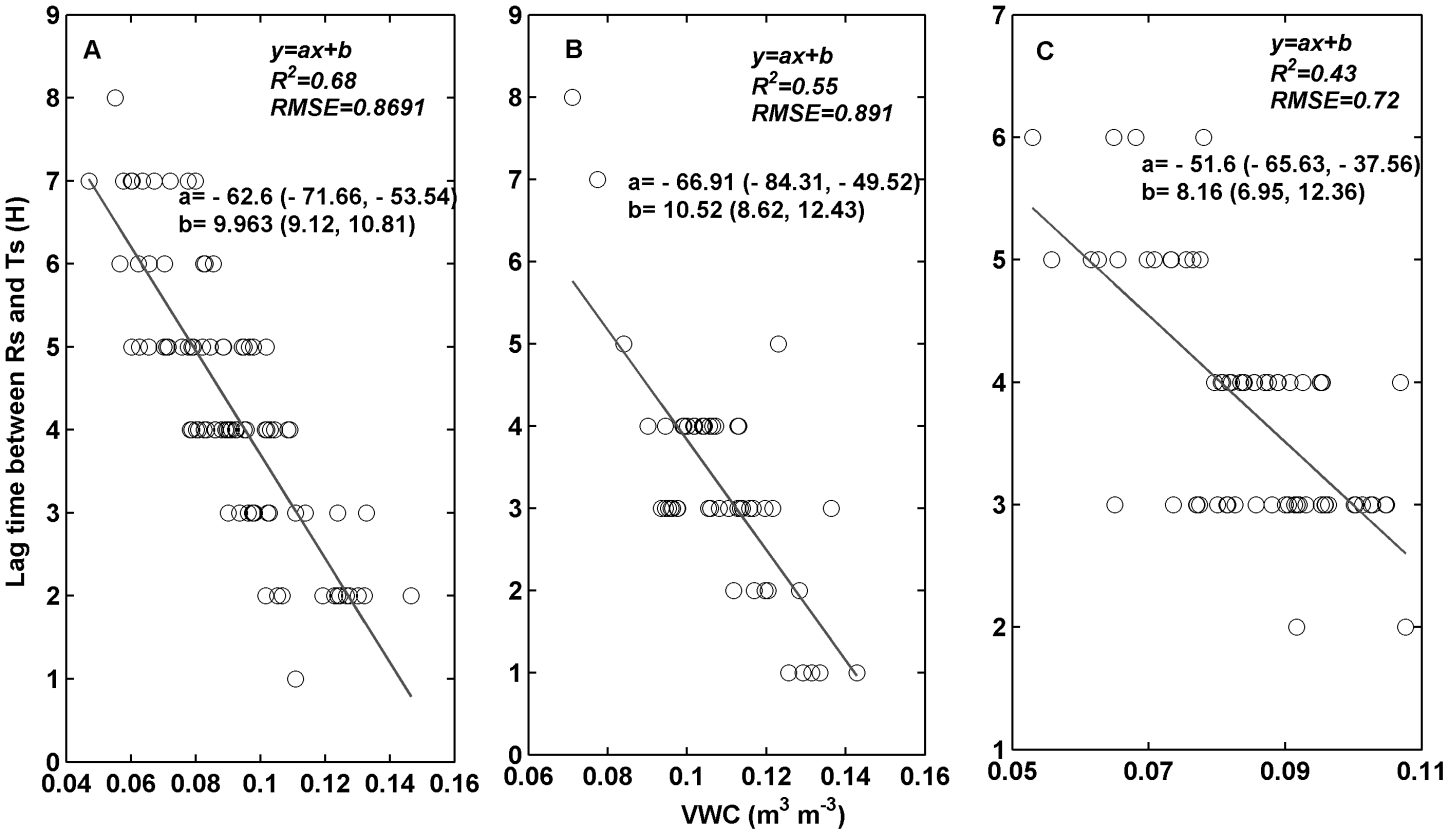
Lag time between soil respiration (*R_s_*) and soil temperature (*T_s_*) over diurnal courses, in relation to soil volumetric water content (*VWC*) in soil crusted with moss (A), lichen (B), and algae (C). The solid line is fitted using linear regression.

Over the course of the diurnal period, *R_s_* (µmol m^−2^ s^−1^) reached its minimum at 6:00 and peaked at around 10:00–11:00 (Fig. 2), and *T_s_* arrived at its minimum at 7:00–8:00 and peaked at 16:00 in the three crusted soils. The diurnal variation of *R_s_* was out of phase with *T_s_*, causing hysteresis between *R_s_* and *T_s_*. The maximum mean lag time between *R_s_* and *T_s_* was 5 h in June in moss-crusted soil, and the minimum mean lag time was 1 h in August in lichen-crusted soil, with *R_s_* peaking earlier than *T_s_* (Table 4). The degree of hysteresis was small in lichen-crusted soil, and large in moss-crusted soil (Table 4). The lag time between *R_s_* and *T_s_* was negatively and linearly correlated with *VWC* in crusted soil (Fig. 3). The lag time was reduced as *VWC* increased. The r values, derived from the data set with synchronized *R_s_* and *T_s_*, were higher than that without synchronization (Table 4).

### 3.2. Seasonal variation in R_s_, T_s_, and VWC

Similar changes in daily mean *T_s_*, *VWC*, and CO_2_ flux (including both daytime and nighttime data) were detected in algae-, lichen-, and moss-crusted soils (Fig. 1). Daily mean *T_s_* was high from June to August, after which it gradually declined (Fig. 1A). No differences were observed in the daily mean nighttime *T_s_* between algae- (18.15±5.61°C, mean ± standard deviation, SD) and lichen-crusted soil (18.14±7.13°C). However, daily mean nighttime *T_s_* in moss-crusted soil (17.45±5.56°C) was significantly lower than that in algae- and lichen-crusted soil (*df* = 2, *F* = 11.92, *P* = 0.013). Daily mean *VWC* sharply increased after each precipitation pulse (Fig. 1B). Daily mean nighttime *VWC* ranged from 0.049 to 0.14 m^3^ m^−3^, 0.057 to 0.16 m^3^ m^−3^, and 0.046 to 0.19 m^3^ m^−3^ in algae-, lichen-, and moss-crusted soil, respectively. Daily mean nighttime *VWC* was significantly higher in lichen-crusted soil (0.104±0.026 m^3^ m^−3^) than in algae- and moss-crusted soils (0.083±0.015 m^3^ m^−3^ and 0.089±0.026 m^3^ m^−3^, respectively) (*df* = 2, *F* = 251.91, *P*<0.001). Daily mean CO_2_ flux varied markedly following the changes in *T_s_* and *VWC*, especially after a rain pulse. Daily mean CO_2_ flux peaked in late July and then generally declined following the decrease in *T_s_* (Fig. 1C). The limiting effect of *VWC* on CO_2_ flux was clear as CO_2_ flux reached its highest value in a quick, sharp response to each rain event and then decreased to pre-rain values (Fig. 1B, C). Daily mean nighttime *R_s_* was significantly different in three crusted soils (*df* = 2, *F* = 56.69, *P*<0.001) with the highest values in lichen-crusted soil (0.93±0.43 µmol m^−2^ s^−1^) and lowest values in algae-crusted soil (0.73±0.31 µmol m^−2^ s^−1^).

**Figure 4:**
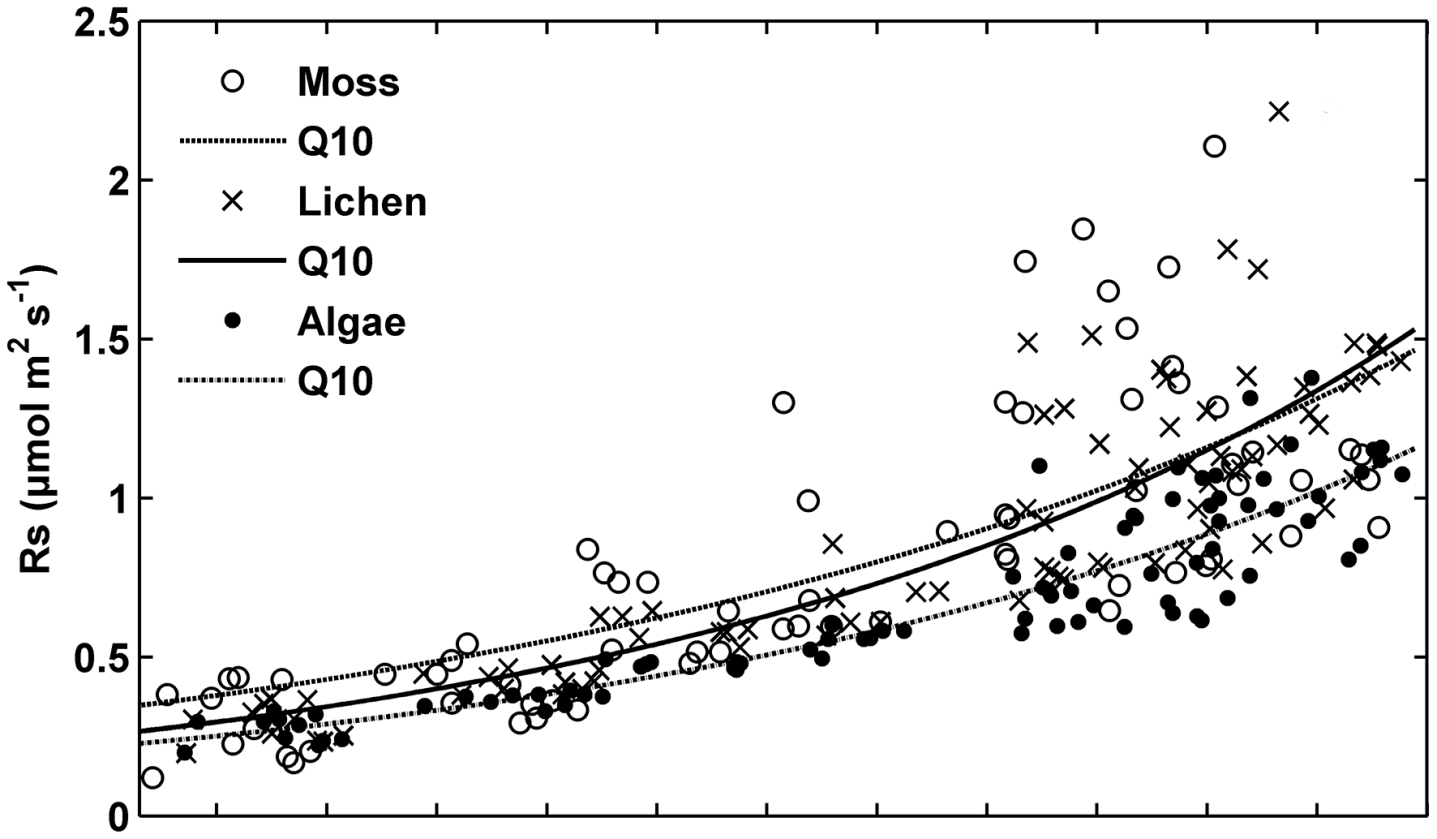
Relationships between daily mean nighttime soil respiration (*R_s_*) and soil temperature (*T_s_*) in algae-, lichen-, and moss-crusted soil.

**Figure 5:**
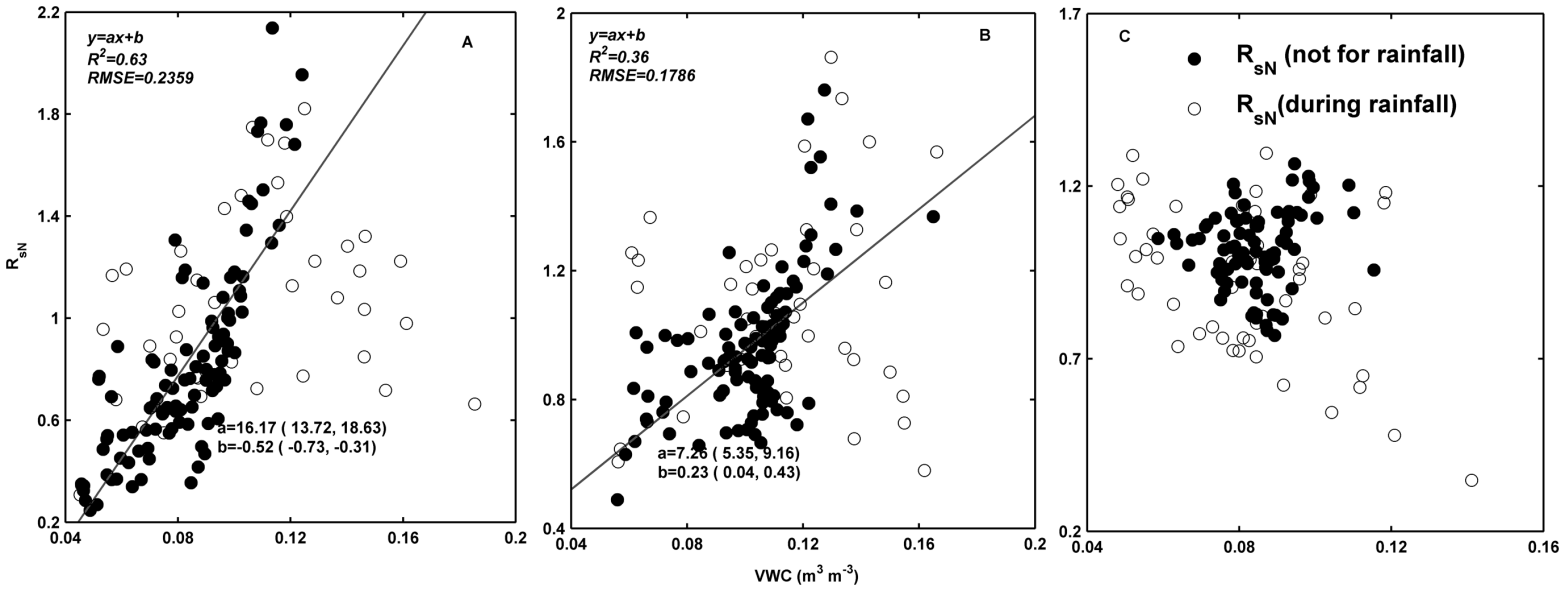
Relationship between daily temperature-normalized mean nighttime soil respiration (*R*_sN_) and soil volumetric water content (*VWC*) at 5-cm depth in moss- (A), lichen- (B), and algae- (C) crusted soil, respectively. *R*_sN_ is the ration of the observed soil respiration (*R_s_*) value to the value predicted by the *Q*_10_ function. The solid line is fitted using linear regression.

Daily mean nighttime *R_s_* was positively related to *T_s_* when *VWC* was higher than 0.075 m^3^ m^−3^ in moss-crusted soil and 0.085 m^3^ m^−3^ in lichen-crusted soil (Fig. 4). There were no differences among the four temperature-response models examined (Table 2). *T_s_* at the5-cm depth explained 82%, 74%, and 51% of the seasonal variation of daily mean nighttime *R_s_* when *VWC* was not a limiting factor in algae-, lichen-, and moss-crusted soil, respectively (Table 2). In algae-crusted soil, however, *R_s_* was controlled by *T_s_* below the *VWC* threshold value (Table 2). As no differences were observed among the temperature-response models, the remainder of the analysis was performed using the *Q*_10_ model. Over the study period, daily mean nighttime *R_s_* normalized using the *Q*_10_ model with *T_s_* at 5 cm depth (*R*_sN_) increased with *VWC*, except in algae-crusted soil (Fig. 5).

The seasonal sensitivity of *R_s_* to *T_s_* (parameter *b* from the *Q*_10_ model in Table 2) were 2.01, 2.13, and 1.97 in algae-, lichen-, and moss-crusted soil, respectively. The long-term basal respiration rate at 10°C (*R_s_*_10_, parameter *a* from the *Q*_10_ model in Table 2) for these same soils was 0.38, 0.46, and 0.55 µol m^−2^ s^−1^.

The bivariate model *Q*_10_-hyperbolic with *T_s_* and *VWC* as independent variables produced higher *R^2^*and lower RMSE values than the other models in lichen- and moss-crusted soil (Table 3). There was no significant difference observed between the temperature-only and the bivariate model in algae-crusted soil (Table 3), and the estimated total C release calculated with the *Q*_10_ model and gap-filled *T_s_* was 123.22 g C m^−2^ in algae-crusted soil (Table 3). The estimated total *R_s_*, as computed using the*Q*_10_-hyperbolic model and gap-filled *T_s_* and *VWC*, was 165.39 and 147.08 g C m^−2^ over the study period in lichen- and moss-crusted soils, respectively. Lichen-crusted soil was the main contributor to this flux among crusted soils during the study period.

## Discussion

### 4.1. Interactive effects of T_s_ and VWC on R_s_

Over the course of the diurnal cycle, there was a significant hysteresis between *R_s_* and *T_s_* (Table 4, Fig. 2). Diurnal hysteresis has been observed in many other ecosystems [16]–[19], [28], [29] and is affected by many physical and biological processes, such as mismatch between the depth of temperature measurement and the depth of CO_2_ production, photosynthetic carbon supply for diurnal *R_s_* [30], wind-induced pressure pumping [31], and different responses of autotrophic and heterotrophic respiration to environmental factors [20]. We observed that the lag time between *R_s_* and *T_s_* was negatively related to *VWC* in the three crusted soils, which is consistent with the finding from the Mu Us desert [19]. The increased lag time at low *VWC* in crusted soils was mainly due to the decoupling of *R_s_* from *T_s_* when *VWC* is low, and which indicate the sensitivity of root and microbial activity to soil moisture. The timing of the diurnal *R_s_* peak is highly sensitive to *VWC*, with progressively earlier peaks as the *VWC* reduces. At low *VWC*, *R_s_* peaks in the early morning due to root and microbial activity may strongly increased with condensation water, resulting to significant hysteresis between *T_s_* and *R_s_* (Fig. 2, Table 4) [19].

The seasonal changes in daily mean nighttime *R_s_* were mainly controlled by *T_s_* (Table 2, Fig. 4). The four temperature-only models performed well with the same *R^2^*. *T_s_* explained 74% and 53% of the variation in *R_s_* when *VWC* was above 0.085 and 0.075 m^3^ m^−3^ in lichen- and moss-crusted soil, respectively, but it was uncorrelated with *R_s_* when *VWC* fell below those thresholds (Table 2). Our observations are in line with those of previous studies in many other ecosystems [8], [21], [28], [32]. Wang et al. [19] reported that *T_s_* explained 76% of the variation in *R_s_* for *VWC* values above 0.08 m^3^ m^−3^, but it was uncorrelated with *R_s_* when *VWC* fell below 0.08 m^3^ m^−3^. Castillo-Monroy et al. [9] found that *R_s_* was controlled by *T_s_* when soil moisture was higher than 11% in microsites dominated by BSCs. Below this level, *R_s_* was driven by soil moisture alone. The decreased *R_s_* under low *VWC* was limited by reduced microbial contact with the available substrate, dormancy and/or death of microorganisms, and substrate supply, which was affected by reduced photosynthesis and drying out of the litter in the surface layer [15], [33].

*R*_sN_ increased with *VWC* and did not show a threshold value in moss- and lichen-crusted soils during the seasonal cycle. Our observation contrasts with the results of previous studies that found a distinct *VWC* threshold [16]. The difference mainly resulted from low *VWC* (0.04–0.16 m^3^ m^−3^) and high soil porosity did not limit CO_2_ transport out of soil and CO_2_ production due to a lack of O_2_.

### 4.2. Differences in R_s_ among biologically-crusted soil types

Daily mean nighttime *R_s_* was significantly different in three types of crusted soils (algae-, lichen- and moss-crusted soil) (*df* = 2, *F* = 56.69, *P*<0.001) with the highest values in lichen-crusted soil and lowest values in algae-crusted soil. This result contrasts with those of other studies in desert ecosystems. Su et al.'s [8] study of Gurbantunggute Desert reported no differences in carbon flux between moss- and lichen/cyanobacteria-crusted soil. The differences in the present study can be explained by the following aspects. It is possible that the lowest *R_s_* in algae-crusted soil resulted from the differences in soil fertility induced by BSCs, total N was significantly lower in algae-crusted soil (0.17±0.09 g kg^−1^) than in lichen- (0.23±0.08 g kg^−1^) and moss-crusted soil (0.28±0.13 g kg^−1^) (unpublished data). In addition, the assemblage of microbial and microfaunal organisms varied in the three crusted soils [10], . The observation of the highest values occurred in lichen-crusted soil was in line with the result conducted in dry condition in the Mu Us desert. The highest values in lichen-crusted soil is mainly due to highest water content and total porosity of lichen layer [10].*T_s_* was significantly lower in moss-crusted soil than in algae- and lichen-crusted soil (Fig. 1). This result is attributed to the darkening of the surface by cyanobacteria and lichens, resulting in greater absorption of solar radiation and a higher surface temperature [39]. *VWC* in lichen-crusted soil was consistently significantly higher than in moss- and algae-crusted soils (Fig. 1). The difference may be attributed to higher dew deposition (soil moisture input by dewfall can be an important mechanism in dryland environment) and water infiltration in lichen-crusted soil than in moss- and algae-crusted soil [37].

The lag time between *R_s_* and *T_s_* differed depending on the type of crusted soil, suggesting that the response of species in biologically-crusted soils to *VWC* was different among crusted types. The timing of the diurnal *R_s_* peak is highly sensitive to *VWC*, with progressively earlier peaks as the soil *VWC* declines [19]. Moss crusts need more *VWC* than lichen and algae crust to achieve metabolic activity [24]. In water stressed ecosystems, algae and lichen can utilize dew and light rainfall that moss are unable to use [24], [27]. Thus the diurnal *R_s_* in moss-crusted soil peaks earlier than algae- and lichen-crusted soils, which lead to significant hysteresis between *R_s_* and *T_s_* in moss-crusted soil. Hysteresis had a smaller impact on lichen-crusted soil than on algae-crusted soil. The result may be partly attributed to the higher water level in lichen- than in algae-crusted soil.

The average *Q*_10_ of 1.83 from three biologically-crusted soil types from June to October is at the lower end of the range of 1.28 to 4.75 from alpine, temperate, and tropical ecosystems across China [38]. The low *Q*_10_ value is attributed to their low levels of soil organic matter, small microbial community, and dry soil conditions [19], [39], [40].The *Q*_10_ of algae-, lichen-, and moss-crusted soil was 1.98, 1.98, and 1.54, respectively. The majority of C associated with BSCs, in the forms of microbial biomass or their secretions [31], [32], is close to or at the soil surface and is directly in contact with small precipitation or dew captured by algae and lichen crusts. However, small amounts of hydration cannot directly reach the soil surface because the soil is covered with moss. The relatively small amounts of hydration in moss-crusted soils result in the lower *Q*_10_ [16], [21], [22], [32].

The effects of *VWC* and *T_s_* on *R_s_* should be considered in carbon cycle models in moss- and lichen-crusted soils. However, we did not find any effect of *VWC* on daily mean nighttime *R_s_* in algae-crusted soil from June to October 2012 (Tables 2, 3). This observation coincided with the result that *R*_sN_ was independent of *VWC* in algae-crusted soil. The independence of *VWC* from *R_s_* in algae-crusted soil may be attributed to the low water requirement of algae for active metabolism [24], [27]. Even a very small hydration event, such as water vapor and dew in the early morning, is sufficient to allow algae to achieve microbial metabolism. Further examination is needed to justify our conclusion regarding the role of *VWC* on algae-crusted soil due to the dew data gap. We used the *Q*_10_-hyperbolic model, with *T_s_* and *VWC* as independent variables, to predict changes in *R_s_*. Using *Q*_10_-hyperbolic model to predict *R_s_* was also reported in a boreal trembling aspen stand [16].

Using temperature-only and *Q*_10_-hyperbolic model, we obtained an approximate estimate of the total amount of C released at each crusted soil via soil respiration of 123.2, 165.4, and 147.1 g C m^−2^ over 5 months studied in algae-, lichen- and moss-crusted soils, respectively. Lichen-crusted soil was the main contributor to the total C released by *R_s_*. We found that total C released by *R_s_* in lichen-crusted soil was 2.5% higher than the mean total C released by *R_s_* (161.4 g C m^−2^, unpublished data) over 5 months, whereas total C released by *R_s_* in algae- and moss-crusted soil were 23.65% and 8.87% smaller than the mean total C released by *R_s_*, respectively. Our results show the importance of BSCs as modulators of *R_s_* in the C release and indicate that we should not ignore their relative contributions to the total C budgets in desert ecosystems.

## Conclusions

Our study showed that *R_s_* was significantly different in three crusted soils with highest values in lichen-crusted soil and lowest values in algae-crusted soil. Lichen-crusted soil was the main contributor to the total C released by *R_s_*. Over the diurnal cycle, *T_s_* exerted dominant control over *R_s_* in the three crusted soils. There was a significant lag between *T_s_* and *R_s_* over the diurnal cycle, and that the lag time increased as *VWC* decreased. Over the seasonal scale, the response of *R_s_* to *T_s_* was regulated by *VWC*, and *R_s_* was uncorrelated with *T_s_* when *VWC* dropped below 0.075 and 0.085 m^3^ m^−3^ in lichen- and moss-crusted soils, respectively. However, *VWC* was not a limiting factor on *R_s_* in algae-crusted soil. Our results indicated that different types of BSCs may affect response of *R_s_* to environmental factors. There is a need to consider the spatial distribution of different types of BSCs and their relative contributions to the total C budgets at the ecosystem or landscape level.
